# Design and Characterization of an RF Applicator for In Vitro Tests of Electromagnetic Hyperthermia

**DOI:** 10.3390/s22103610

**Published:** 2022-05-10

**Authors:** Riccardo Ferrero, Ioannis Androulakis, Luca Martino, Robin Nadar, Gerard C. van Rhoon, Alessandra Manzin

**Affiliations:** 1 Istituto Nazionale di Ricerca Metrologica (INRIM), 10135 Torino, Italy; l.martino@inrim.it (L.M.); a.manzin@inrim.it (A.M.); 2 Department of Radiotherapy, Erasmus MC Cancer Institute, University Medical Center, 3015 GD Rotterdam, The Netherlands; r.nadar@erasmusmc.nl (R.N.); g.c.vanrhoon@erasmusmc.nl (G.C.v.R.); 3 Department of Radiation Science and Technology, Delft University of Technology, 2629 JB Delft, The Netherlands

**Keywords:** thermal therapies, electromagnetic hyperthermia, RF applicator, TEM mode, coaxial cable, electromagnetic modelling, thermal modelling, temperature measurements, phantoms

## Abstract

The evaluation of the biological effects of therapeutic hyperthermia in oncology and the precise quantification of thermal dose, when heating is coupled with radiotherapy or chemotherapy, are active fields of research. The reliable measurement of hyperthermia effects on cells and tissues requires a strong control of the delivered power and of the induced temperature rise. To this aim, we have developed a radiofrequency (RF) electromagnetic applicator operating at 434 MHz, specifically engineered for in vitro tests on 3D cell cultures. The applicator has been designed with the aid of an extensive modelling analysis, which combines electromagnetic and thermal simulations. The heating performance of the built prototype has been validated by means of temperature measurements carried out on tissue-mimicking phantoms and aimed at monitoring both spatial and temporal temperature variations. The experimental results demonstrate the capability of the RF applicator to produce a well-focused heating, with the possibility of modulating the duration of the heating transient and controlling the temperature rise in a specific target region, by simply tuning the effectively supplied power.

## 1. Introduction

Hyperthermia is a potent sensitizer to radiotherapy and chemotherapy [1,2]. Its effectiveness on tumor treatment has been demonstrated in in vitro studies on cell cultures, in preclinical tests conducted on animals and also in clinical trials [3,4]. Despite its current use in clinics, as an efficacious and safe therapy, the complete understanding of its mechanism of action on cells and tissues is still a matter of scientific debate. Other issues are related to the fulfillment of requirements such as reproducibility and control of temperature distribution in the target region. Several efforts have been made to improve heating uniformity and target specificity while minimizing invasiveness in clinics, also comprising the implementation of treatment planning techniques [5,6,7]. Indications on how enhancing therapeutic outcomes have been provided by a large number of preclinical studies, but the weak reproducibility and comparability of results have made standardization actions necessary [8].

Regarding studies in vitro, several research groups have attempted to perform thermal dosimetry on cells when exposed to hyperthermia, as well as to better quantify the cytotoxic effects of hyperthermia when combined with other treatments, such as radiotherapy [9,10,11,12] or chemotherapy [13]. In the past, the studies were conducted on 2D cell cultures [10], which represent a simple in vitro model, poorly mimicking the in vivo conditions. Recently, 3D tumor spheroids and organoids have been emerged as in vitro models able to emulate the structural organization and cellular assembling of human solid tumors, providing better insight also for hyperthermia tests [14].

In most of the in vitro studies of hyperthermia biological effects, cell cultures are conventionally heated using warm water baths [14,15,16]; with this approach the in vitro system is heated through convection from outside to inside. In preclinical research, many hyperthermia solutions have been proposed, also addressed by in silico modelling. These include cold-light sources, near-infrared laser light, focused ultrasound (US) applicators, capacitive devices, microwave (MW) and radiofrequency (RF) electromagnetic (EM) systems, and magnetic nanoparticles excited by RF magnetic fields [8,17,18,19,20,21,22,23,24]. Some of these techniques mainly guarantee a surface power deposition, leading to a preferential heating of the external parts of the body (e.g., cold-light sources, near-infrared laser light, MW radiation), while the others can be adapted for both superficial and deep heating. For the latter, the heat control and focusing are more critical, nevertheless many hyperthermia applicators have been designed to provide an inside-out heating and successfully applied in clinical trials to treat deep-seated tumors [25,26,27].

The way of delivering heat (from outside to inside or vice versa) might impact differently also on the effects induced in cell cultures, especially for 3D systems, where a precise power release is required to improve thermal dosimetry and correlation with the biological response. Focusing on EM fields, one way to produce a controllable power deposition is through a phased antenna array [28], which exploits constructive interference of an array of antennas to obtain a well-focused heating and tune penetration depth. This method offers flexibility thanks to focus adaptability, however it needs careful design of each of the interfering antennas, in terms of phase and amplitude.

A simpler strategy for a controlled and internal heat release is represented by the coaxial transverse electromagnetic (TEM) applicator proposed by Lagendijk in [29]. This is based on an open ended coaxial air line with a hollow inner conductor; at the aperture, where the target region is placed, the EM field has a radiative circumferential configuration. The main advantage of such applicator is the possibility of adjusting the penetration depth and size of the heating focus, always located on the central longitudinal axis, by simply tuning the aperture width [29,30,31]. Another advantage is the possibility of operating in a wide range of frequencies.

In this paper, we readapt and rescale the prototype proposed by Lagendijk to develop an RF hyperthermia applicator working at 434 MHz, suitable for in vitro studies on 3D tumor spheroids and organoids, and potentially for preclinical tests on small animals, such as mice. The RF applicator is designed with the aid of combined EM and heat transfer modelling, and tested on tissue-mimicking phantoms, performing a wide campaign of measurements of the spatial-temporal distribution of the temperature, as a function of the effectively supplied power. The final objective is to obtain a well-focused heating and a controllable and uniform temperature rise in a target region, i.e., a 1 cm^3^ volume of interest (VOI) within the phantom.

## 2. Materials and Methods

### 2.1. Numerical Modelling

To support the design of the RF applicator, in terms of optimal dimensions, power deposition and heating efficiency, we perform EM simulations followed by heat transfer modelling. Both types of simulations are carried out by means of the advanced computation platform Sim4Life (v6.2, ZMT, Zurich, Switzerland) [32]. A non-uniform grid is used to discretize the 3D domain under study Ω, which is a rectangular prism including the whole volume of the applicator, and expanded by 17.3 cm in each direction, so that its boundary ∂Ω does not influence the electric field generated by the applicator. The grid resolution varies between 0.25 mm, in the area occupied by the phantom, and 30 mm at the computational domain boundary ∂Ω. A geometry resolution of at least 2 mm is guaranteed in the whole applicator volume.

#### 2.1.1. Electromagnetic Simulations

Within the Sim4Life framework, the EM field is calculated by solving Maxwell’s equations:

(1) $$\left\{ \begin{array}{l} \frac{\partial H}{\partial t}=-\frac{1}{\mu }\nabla \times E\\ \frac{\partial E}{\partial t}=\frac{1}{\varepsilon }\nabla \times H-\frac{\sigma }{\varepsilon }E \end{array}\right.$$

with a Finite-Difference Time-Domain (FDTD) method, using a second-order FD scheme [33]. In Equation (1) **H** and **E** are the magnetic and electric field vectors, respectively, *μ* is the magnetic permeability, *ε* is the permittivity and *σ* is the electrical conductivity of the medium. **H** and **E** are integrated on a staggered mesh and time-updated with a leap-frog scheme. On ∂Ω, Absorbing Boundary Conditions are imposed.

All the metallic media are modelled as perfect electric conductors (PECs), while the other material electrical properties are assigned according to Table 1. In particular, high-density polyethylene (HDPE) is used for the dielectric medium, polyvinyl chloride (PVC) for the phantom holder and an agar gel for the phantom, with the electrical properties of a water-based tissue, such as muscle [34,35].

As for the power supply, we consider a harmonic edge source with a frequency of 434 MHz, a voltage of 1 V and a load of 50 Ω between the inner and outer conductors of the coaxial cable.

#### 2.1.2. Thermal Simulations

For the thermal simulations, we use the transient solver within the Sim4Life platform that implements the heat transfer equation in solids, namely:

(2) $$\{\begin{array}{l} \rho C_{p}\frac{\partial T}{\partial t}=\nabla \cdot k\nabla T+P_{\mathrm{loss}}\\ {T|}_{\partial \Omega }=T_{\mathrm{ext}} \end{array},$$

where *T* is the temperature, *ρ* is the medium density, *C*_p_ is the heat capacity, *k* is the thermal conductivity and *P*_loss_ is the power loss density resulting from the EM simulations. Dirichlet boundary conditions are imposed at ∂Ω, where *T* is fixed to the room temperature *T*_ext_, measured before the experiments. The initial temperature for all the materials is also set at *T*_ext_.

Since the metal walls of the RF applicator are highly thermally conductive and very thin compared to the other component size, they are excluded from thermal simulations. The thermal properties of the other materials are listed in Table 1.

### 2.2. Characterization and Measurement Set-Up

#### 2.2.1. Reflection Measurements

The power reflection coefficient (S11) of the RF applicator is measured under operation conditions (with the agar-gel phantom inside) using a two-port vector network analyzer (VNA), Model R&S^®^ZNC3, Rhode&Schwarz (Munich, Germany). The VNA, which can operate in a range from 9 kHz to 3 GHz, is matched at 50 Ω using an input power of 10 dBm.

#### 2.2.2. Power Supply and Monitoring

To supply the necessary power, the RF applicator is connected via a 50 Ω coaxial cable to a 434 MHz power generator [36]. The input and reflected powers are measured with two power meters, Model PWR-4GHS Mini Circuits (Brooklyn, NY 11235, USA), connected via a bidirectional coupler (−50 dB coupling). The readout of the power meters is used to evaluate the effectively supplied power and the RF applicator efficiency.

#### 2.2.3. Phantom Preparation and Characterization

The hyperthermia performance of the RF applicator is tested on phantoms made of agar gel, specially doped to mimic the electrical properties of a water-based tissue, such as muscle [34,35]. The phantoms are produced with the following recipe: 1000 g of distilled water is mixed with 50 g of agar-agar and 5.7 g of NaCl to tune the electrical properties. The mixture is stirred and heated up to 90 °C. The resulting solution is poured into the dedicated sample holders to produce cylindrical phantoms with diameter of 3 cm and height of 10 cm. All the phantoms are made from the same batch of agar solution to keep the properties as homogeneous as possible among them. The resulting electrical properties are measured with a Dielectric Assessment Kit (DAK) 12 probe (4 MHz–3 GHz), SPEAG (Zurich, Switzerland), which also includes the software for the data acquisition; phantoms with a larger diameter have been specifically developed to perform this characterization.

The phantoms are subsequently prepared for the temperature measurements. The ones used for the measurement of the time evolution of the temperature are opportunely modified to accommodate a set of fiber-optic thermometers. The ones used for the temperature mapping with an infrared (IR) thermal imaging camera are cut lengthwise along one of the central planes (split phantoms). Each phantom entirely fills the containing PVC tube, which tightly fits inside the aperture to avoid air gaps.

#### 2.2.4. Measurement of Temperature Time Evolution

The temperature of the agar-gel phantom during the hyperthermia session is monitored by means of an array of fiber-optic thermometers, Model Evolution, FISO (Quebec City, QC, Canada), which can operate under the application of the RF field without EM interference and contribution to the sample heating. Real-time acquisition of the temperature of the phantom is therefore possible with the RF field switched on. This is performed with a set of three fiber-optic sensors (±0.1 °C accuracy between +20 °C and +50 °C), having multiple measuring points along their length and placed at regular intervals along the phantom radial direction. The room is climate controlled, and the ambient temperature is kept to 21.5 ± 1 °C for the whole duration of the experiments.

#### 2.2.5. Measurement of Temperature Spatial Distribution

To map the temperature spatial distribution inside the split phantom, thermal images of the phantom central plane are acquired by means of an IR thermal camera, Model FLIR T1020 (Wilsonville, ON, USA). The accuracy of the thermal camera was previously tested in a controlled laboratory environment. A flat homogenous phantom was left for 1 h at room temperature under stable and controlled conditions, to reach thermal equilibrium. The thermal camera was placed at a fixed distance of 1 m from the phantom surface to acquire consecutive thermal images, after adapting the lens focus. The temperature measured on the phantom surface matched the ambient temperature with a standard deviation of 0.2 °C. Hence, an accuracy of ±0.2 °C is expected under the same laboratory conditions.

## 3. Design of the RF Applicator

**Figure 1:**
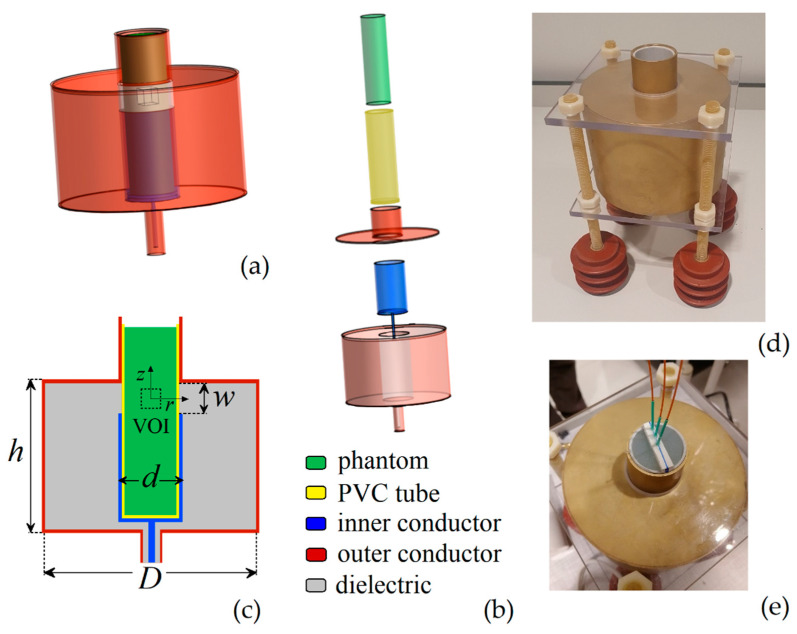
Schematics of the RF applicator: (**a**) 3D view with mounted components; (**b**) main components with representation of insertion; (**c**) longitudinal cross-section with indication of the main parameters, i.e., *d* is the outer diameter of the inner conductor, *D* is the inner diameter of the outer conductor, *w* is the aperture width and *h* is the external height of the outer conductor. The dashed line indicates the target region or volume of interest (VOI). Pictures of the RF applicator: (**d**) frontal view, with insulating and mechanical support; (**e**) top view showing the phantom with the fiber-optic thermometers.

The RF applicator is designed according to the TEM mode in a coaxial cable, rescaling the system proposed in [29] to obtain an applicator suitable for in vitro tests and, potentially, for preclinical tests on small animals. As schematized in Figure 1a,b, this consists in an outer cylindrical conductor (in red) connected to the metallic shield of the feeding coaxial cable and a hollow inner cylindrical conductor (in blue), connected to the coaxial cable core. The inner conductor is engineered to irradiate the EM field in a specific target region, centered in the aperture, where the phantom (in green) is positioned after being inserted in a PVC tube (in yellow). Within the aperture, the EM field is circumferential and radiative, with an expected heating focus in correspondence of the aperture center. The space between the two conductors is filled with a dielectric medium (in light grey), made of HDPE.

### 3.1. Parametric Analysis

As described in Section 2.1.1., the parametric analysis for the design of the RF applicator is performed via EM numerical modelling. The shape and size of the final prototype derives from the following requirements:

(1) the aperture has to be properly dimensioned to guarantee the accommodation of standard vials for in vitro tests on 3D cell cultures;
(2) the heating focus has to be positioned within a cubic target region of 1 cm^3^ volume (VOI), where a homogeneous power deposition should be obtained (schematic in Figure 1c);
(3) under operating conditions, the applicator should match as close as possible a load with an impedance *Z* of 50 Ω, in order to minimize reflections and avoid the need of a matching network for its operation.

Once selected the material for the dielectric medium within the coaxial system (with relative permittivity *ε*_r,dielectric_), the fulfillment of the third requirement imposes a constraint on the ratio of the outer conductor diameter *D* to the inner conductor diameter *d*, according to the following simplified relationship:

(3) $$Z=\frac{138\log_{10}\left(\frac{D}{d}\right)}{\sqrt{\varepsilon_{r,\mathrm{dielectric}}}}.$$

The chosen dielectric medium is HDPE. Since the main objective is the control of the power deposition within the target region, the parameters first analyzed are *d* and the aperture width *w*; then parameter *D* will be tuned according to Equation (3). We test 3 values for *d* (2.2 cm, 3.5 cm and 4.3 cm) and 4 values for *w* (0.7 cm, 1.2 cm, 1.7 cm and 2.2 cm), for a total of 12 combinations, fixing the height *h* of the outer conductor to 8 cm. The values of *d* are chosen on the basis of the standard dimensions of the PVC tubes for holding the phantoms, whose dimensions are adapted in order to completely fill the aperture.

**Figure 2:**
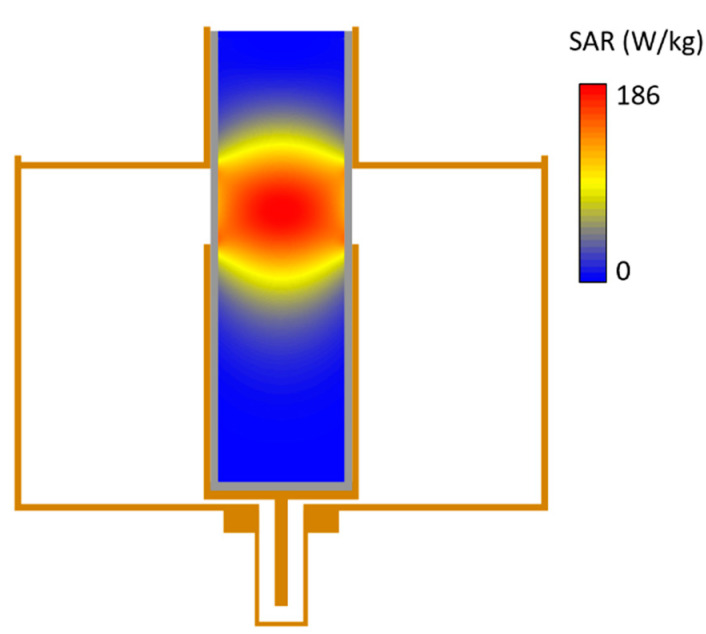
Spatial distribution of the specific adsorption rate (SAR) in the phantom, calculated over a longitudinal cross-section of the RF applicator, for parameters *d* = 3.5 cm, *D* = 11.7, *w* = 1.7 cm and *h* = 8 cm (see definition in Figure 1c).

For each combination of parameters, we perform EM simulations and evaluate the power deposited in the target region. As an example, Figure 2 reports the spatial distribution of the specific adsorption rate (SAR) over a longitudinal cross-section of the RF applicator, for *w* = 1.7 cm, *d* = 3.5 cm and *D* = 11.7 cm (dimensions of the built prototype, as described in the following Sub-section). It is clear that the heating focus is placed in correspondence of the center of aperture, where a strong uniformity of the power deposited is found, with a more rapid decay along the longitudinal direction than the radial one.

**Figure 3:**
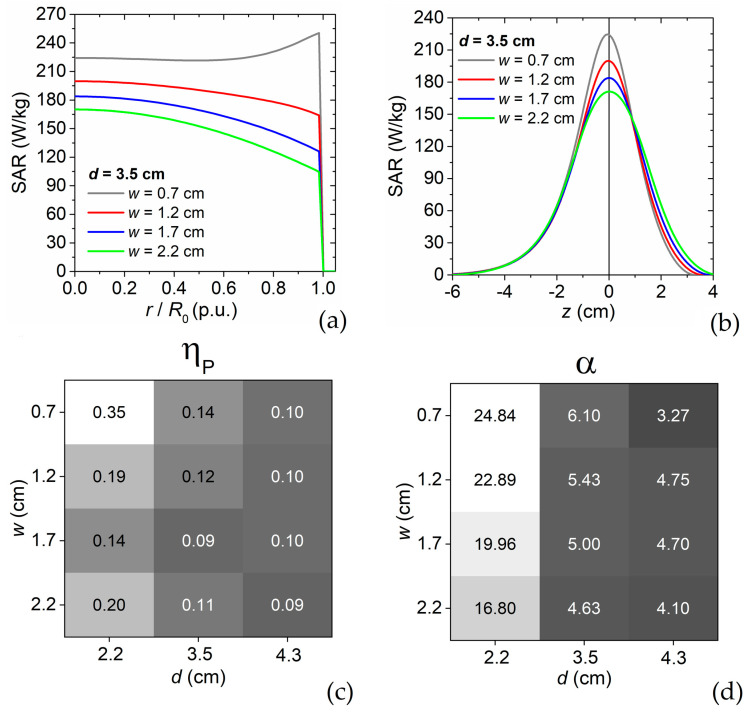
Spatial variation of SAR in the phantom calculated along the (**a**) radial and (**b**) longitudinal axes depicted in the schematic of Figure 1c. The calculations are performed for variable values of the aperture width *w*, fixing the diameter *d* of the inner conductor to 3.5 cm. (**c**) Grey-scale maps (light grey corresponds to the highest values) of the (**c**) coefficient of power heterogeneity *η*_P_ and (**d**) percentage *α* of power deposited in the VOI, as a function of parameters *w* and *d*.

The behavior of the RF applicator as a function of *w* is illustrated in Figure 3, which shows the spatial variation of the SAR in the phantom, calculated along the radial (Figure 3a) and longitudinal (Figure 3b) axes, for all the considered values of *w*, fixing *d* to 3.5 cm. For the lowest value of *w* (0.7 cm), the RF applicator has a well distinctive behavior, with a power increase at the phantom boundary. For the other values of *w*, the power peak is located at the VOI center, becoming progressively sharper on the radial direction and wider on the longitudinal axis as *w* increases. A similar behavior of the power deposition spatial distribution can also be found for the other values of *d*; the relative variations along the radial and longitudinal directions are reported in Figure S1 of the Supplementary Materials.

The results obtained for the different combinations of parameters *d* and *w* are compared considering as metrics the coefficient of heterogeneity *η*_P_ and the percentage *α* of power deposited in VOI (i.e., the 1 cm^3^ target region). The coefficient of power heterogeneity *η*_P_ is calculated as follows:

(4) $$\eta_{P}=\frac{P_{5}-P_{95}}{P_{5}},$$

where *P*_5_ and *P*_95_ are the power deposition densities reached within at least 5% and 95% of the VOI, respectively; lower values of *η*_P_ correspond to better performance of the RF applicator in terms of uniformity of the power density spatial distribution. The percentage *α* of the power deposited in the VOI is an indicator of the efficiency of the RF applicator, providing a measure of the power deposited in the target region with respect to the total supplied power.

The obtained results are resumed in Figure 3c,d for *η*_P_ and *α*, respectively. A non-monotonic behavior of *η*_P_ versus *w* is found when *d* is equal to 2.2 cm and 3.5 cm, with the highest level of uniformity achieved for *w* = 1.7 cm. The increase in *d* leads to a reduced influence of the aperture width; when *d* = 4.3 cm, the role of *w* becomes negligible and the values of *η*_P_ result very low. The worst uniformity is found when *d* = 2.2 cm and *w* = 0.7 cm. Regarding *α*, when *d* is equal to 2.2 cm and 3.5 cm the power percentage deposited inside the VOI is higher for the narrowest apertures, while a practical opposite behavior is found for *d* = 4.3 cm.

In summary, inner conductors with larger diameters and wider apertures guarantee a greater uniformity of power deposition at the expense of the applicator efficiency.

### 3.2. Final Prototype

For the implementation of the final prototype we choose brass for the metallic components and we set the inner conductor diameter *d* at 3.5 cm and the aperture width *w* at 1.7 cm. This choice is dictated by the need of limiting the dispersion of power in non-target regions maintaining an overall good level of power homogeneity in the VOI (see Figure 3c,d). According to Equation (4) and the constraint on *Z*, it results in an outer conductor diameter *D* of 11.7 cm, which represents a good compromise in terms of practical use and reduced encumbrance.

A picture of the front view of the built RF applicator is reported in Figure 1b, where it is also possible to see the four support insulators (in epoxy resin) and the components introduced to guarantee mechanical stability, i.e., two polycarbonate plates, placed at the bottom and top, and fixed with plastic nuts and bolts. A standard N female RF coaxial connector is soldered to the bottom of the applicator. The top view of the applicator is shown in Figure 1c, where we can also see the phantom within the PVC tube equipped with the fiber-optic thermometers.

## 4. Results from Characterization of the Experimental Set-Up

Before the investigation of the heating efficiency, we measure the electrical properties of the used phantoms and the power reflection coefficient (S11) of the built RF applicator under operation conditions.

### 4.1. Phantom Electrical Properties

**Figure 4:**
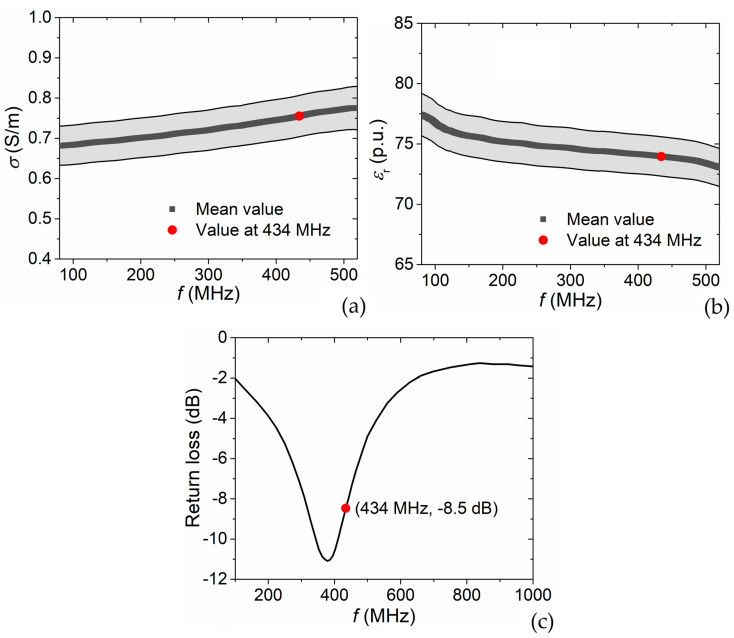
Frequencies dependencies of phantom (**a**) electrical conductivity and (**b**) relative permittivity, measured with a coaxial probe (mean values and standard deviations are reported). (**c**) Frequency dependence of the return loss of the RF applicator, measured under operation conditions (phantom within the aperture).

The phantom electrical characterization is performed according to the description in Section 2.2.3. The frequency dependencies measured for the electrical conductivity *σ* and relative permittivity *ε*_r_ are illustrated in Figure 4a,b, respectively, considering a frequency range between 80 MHz and 520 MHz, with a sampling interval of 1 MHz. The measurements have been repeated 5 times changing the contact point between the probe and the phantom to reduce the effect of possible local disturbances; for each frequency value, we estimate mean value and standard deviation.

For the operation frequency of 434 MHz the measured mean values of *σ* and *ε*_r_ are 0.755 S/m and 74, respectively, well mimicking the electrical properties of a water-based tissue, such as muscle [34,35]. These values are considered as material parameter inputs for the EM simulations, as reported in Table 1.

### 4.2. Power Reflection Coefficient

The power reflection coefficient (S11) of the RF applicator is measured in the frequency range between 100 MHz and 1 GHz, as described in Section 2.2.1. As can be seen in Figure 4c, S11 decreases significantly within the interval 300–500 MHz, reaching a magnitude of −8.5 dB at 434 MHz. This data corresponds to a return loss around 15%, which indicates adequate matching with the 50 Ω impedance of the coaxial cable. Therefore, the returned power is sufficiently low to allow the connection of the applicator directly to the power source without the need of a matching network.

## 5. Results from Characterization of Heating Efficiency

The heating efficiency of the built RF applicator is characterized by performing two types of thermometric measurements. As described in Section 2.2.4., one aims at characterizing the temporal response of the system as a function of the effectively supplied power, monitoring the heating and cooling transients in a set of points within the phantom. As introduced in Section 2.2.5., the second one is finalized to the characterization of the heating focus, through the measurement of the temperature spatial distribution at the end of the heating transient.

### 5.1. Heating-Cooling Transients

**Figure 5:**
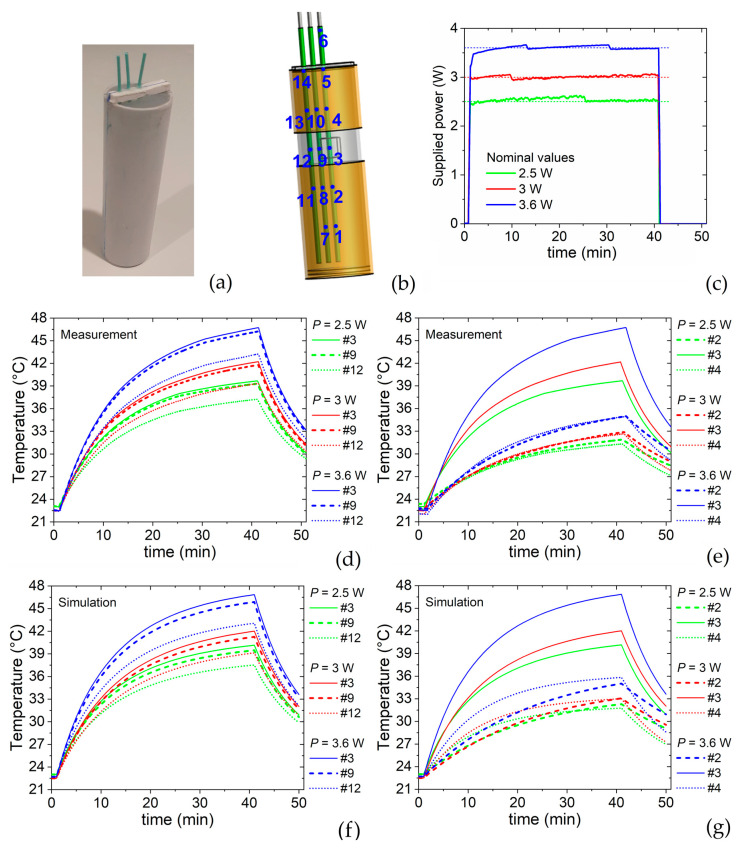
(**a**) Picture of the phantom with the three fiber-optic thermometers arranged along the radial direction. (**b**) Schematics of the phantom, showing the position of the VOI and of the 14 measurement points distributed along the fiber-optic thermometers. (**c**) Time evolution of the effectively supplied power for three power nominal values (2.5 W, 3 W and 3.6 W, as indicated by dashed lines). Heating-cooling transients as a function of supplied power levels: measured time-evolution in points (**d**) #3, #9 and #12, and (**e**) #2, #3 and #4; simulated time-evolution in points (**f**) #3, #9 and #12, and (**g**) #2, #3 and #4.

The RF applicator is tested to evaluate the temperature reached after a fixed time interval, as a function of the effectively supplied power. The objective is to find the appropriate level of power able to guarantee the reaching and maintaining (for an adequate time interval) of a predefined target temperature in the VOI. To this aim, we monitor the temperature along time with three fiber-optic sensors, placed at 0.5 cm intervals along the radial direction, i.e., at zero, 0.5 cm and 1 cm distance from the central axis of the cylindrical phantom (as shown in Figure 5a). Each fiber-optic sensor, which has multiple measuring points along its length with adjacent distance of 2 cm, is inserted in the phantom, so that one of the measuring points (the one numbered as #3) is positioned in correspondence of the VOI center (see the schematic in Figure 5b).

As a target temperature in the VOI center, we chose 43 °C. For the determination of the required power levels, the experiments are conducted by supplying the applicator for 40 min, while keeping the effectively supplied power (input power minus reflected power) as close as possible to the nominal values of 2.5 W, 3 W and 3.6 W (Figure 5c). Figure 5d reports the time evolution of the temperature recorded at the measurement points #3, #9 and #12, which are distributed along the radial axis with origin in the VOI center. The maximum temperature, measured in point #3, is equal to 39.7 °C, 42.2 °C and 46.7 °C for the power levels of 2.5 W, 3 W and 3.6 W °C, respectively. A limited reduction in temperature is found in point #9, located at the VOI boundary, as a proof that within the VOI the temperature is strongly uniform. A decrease in the order of 7.5% is recorded in point #12, with respect to the temperature at the VOI center, for the power level of 3.6 W. After 40 min, the power is no more supplied and the temperature rapidly decreases, especially within the VOI. For the 3.6 W nominal power case, a temperature of 33 °C is measured in point #3, 10 min after the power stopping.

The temperature variation along the phantom central axis is illustrated in Figure 5e, which reports a comparison of the temperature time evolutions recorded in points #2, #3 and #4 for all the power nominal values. At vertical distances of 2 cm from the VOI center (moving both downward and upward outside the VOI), we observe a temperature reduction around 14% and 20–21%, for the power levels of 3.6 W and 2.5 W, respectively. The temperature time evolutions measured in the remaining points are shown in the Supplementary Material in Figure S2.

The heating-cooling transients are also simulated for comparison and mutual validation. Given the uncertainty on material properties and position of the fiber-optic sensors, a good fit to experiment is found along the radial axis with origin in the VOI center. As demonstrated by the comparison of Figure 5d,f, a very similar behavior of measured and simulated thermal curves is indeed found for the entire time interval in measuring points #3, #9 and #12. However, along the vertical axis, the simulated thermal curves show slight differences from the measured ones, especially during the heating transient; the measured and simulated peak temperatures differ less than 0.5 °C (comparison of Figure 5e,g). The maximum discrepancy, resulting lower than 1 °C, is observed for the power level of 3.6 W in correspondence of measuring point #4, as a possible consequence of its closer proximity to the external environment and a greater influence of the heat transfer conditions, not perfectly known.

**Figure 6:**
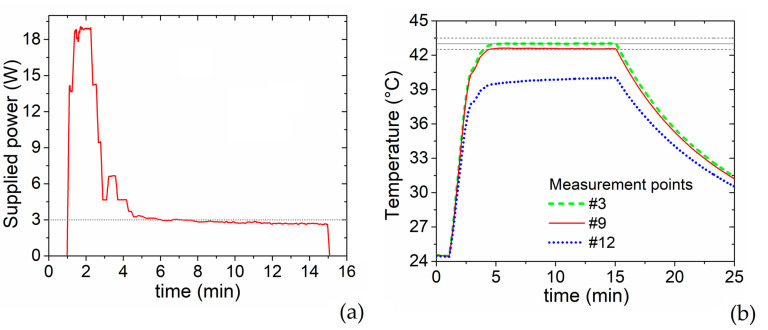
(**a**) Time evolution of the power effectively supplied to the RF applicator to perform steady-state measurements at 43 °C. (**b**) Consequent heating-cooling transients, measured in points #3, #9 and #12. The target temperature of 43 °C is indicated with a solid dark grey line, while the dashed lines indicate a ±0.5 °C difference from the target temperature.

From the results in Figure 5d,e, it is clear that an effectively supplied power of 3 W is sufficient to maintain the target temperature of 43 °C. This is well demonstrated by Figure 6b, where we report the time evolution of the temperature, monitored in points #3, #9 and #12, when the effectively supplied power has the waveform depicted in Figure 6a. In particular, a high power (in the order of 18 W) is provided for the first 2 min to reach more rapidly the target temperature, then followed by a steep decrease to values between 3 W and 3.4 W, maintained for 10 min to allow steady-state measurements. As expected, these power levels enable to keep the temperature around the target value (Figure 6b); in particular, during the 10 min after the preheating phase the temperature reaches a mean value of 43.01 ± 0.01 °C (standard deviation) in the VOI center (point #3) and 42.58 ± 0.02 °C on the VOI boundary (point #9).

The above results prove that the RF applicator is able to guarantee a strong uniformity of the temperature in the VOI and a stable maintenance of the target temperature, achieved with a supplied power tuning.

### 5.2. Heating Focus

The extension of the heating focus is characterized by measuring the spatial distribution of the temperature inside the split phantom (Figure S3a in the Supplementary Materials), after supplying the RF applicator with a 5 W power for 5 min. Then, the split phantom is removed from the sample container, opened and an image of the temperature spatial distribution is immediately taken with the IR thermal camera. The temperature acquisition is performed within 3 min from power off.

**Figure 7:**
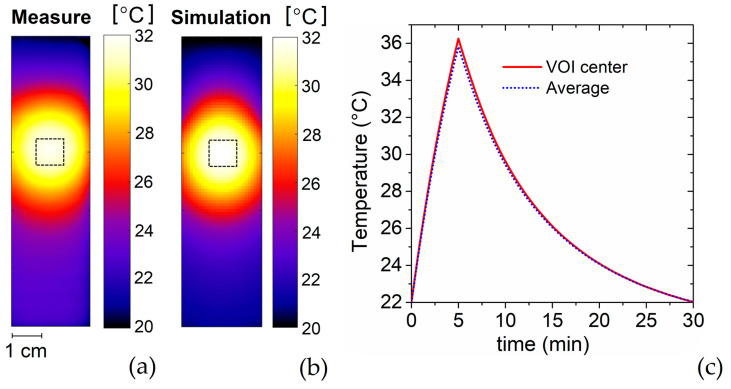
(**a**) Spatial distribution of temperature measured on the central longitudinal plane of the split phantom, 3 min after heating the phantom with a 5 W power for 5 min; the data are acquired with IR thermal camera. (**b**) Simulated spatial distribution of temperature. (**c**) Calculated time evolutions of the average temperature in the VOI and of temperature value in correspondence of the VOI center.

The measured temperature map is shown in Figure 7a (the one directly recorded by the IR thermal camera is shown in the Supplementary Materials in Figure S3b); the calculated temperature spatial distribution is reported in Figure 7b for comparison and mutual validation. The two maps show a very good agreement with very similar temperature profiles; the differences in the peak values are expected to be within the margin of errors of the instrument and the numerical approximation introduced in the simulations. For both the measured and calculated maps, the maximum temperature, recorded in correspondence of the VOI center, is around 32 °C, value that can be varied by tuning the effectively supplied power. This temperature is reached according to the transient reported in Figure 7c, for the simulation case. The weak differences between the temperature calculated in the VOI center and its average over the entire VOI prove the capability of the applicator to guarantee a strongly unifom spatial distribution of the temperature in the target region. In particular, within the VOI the coefficient of temperature heterogeneity *η_T_* results equal to 0.06, assuming the following definition for *η_T_*:

(5) $$\eta_{T}=\frac{T_{5}-T_{95}}{T_{5}},$$

where *T*_5_ and *T*_95_ are the temperatures reached within at least 5% and 95% of the VOI, respectively. The low value of *η_T_* demonstrates the ability of the RF applicator to generate not only a controlled and uniform power deposition in the VOI, but also a homogeneous and well-targeted temperature raise. Outside the VOI, the temperature decays quite rapidly; a reduction of 50% in the temperature raise is observable at distances of 0.5 cm from the VOI boundary. All these features make the built RF applicator a device that can be potentially used for standardizing hyperthermia measurements on cell cultures, as well as for calibrating other hyperthermia setups, such as the ones used in magnetic hyperthermia, where the control of temperature is even a more critical issue [37,38].

## 6. Conclusions

The accurate thermal dosimetry in EM hyperthermia is still an open issue. In this paper we have designed, optimized and characterized an efficient and easy-to-operate RF applicator working at 434 MHz that allows to obtain well controlled heat delivery and temperature rise. This can be used as a calibrated setup in hyperthermia tests on tissue-mimicking materials, 3D cell cultures and tumor organoids, and potentially in the treatment of tumors in small animals typically used in preclinical tests, e.g., 20–25 g mice.

Among the several advantages, the working principle of the developed RF applicator enables to guarantee a great reproducibility in terms of power deposition and temperature increase in the VOI, a target region with 1 cm^3^ volume located at the center of the heating focus. The above results have been documented by a campaign of measurements of the temperature time evolution and spatial distribution, performed on phantoms with muscle-like tissue properties. In addition to reproducibility, with the developed applicator it is possible to achieve a strong uniformity and stability in the VOI for power deposition as well as temperature increase, which can be controlled in a quite extended range by tuning the supplied power. A difference in temperature lower than 0.5 °C is recorded between the VOI center and the VOI boundary, with a rapid decrease outside (50% in 0.5 cm), demonstrating the high heat focusing of the RF applicator.

The tuning of the supplied power also enables to precisely modulate the duration of the heating transient and the rate of temperature change over time. Moreover, the applicator requires very low power levels to reach target temperatures suitable for hyperthermia. Return loss values between 7.5 dB and 10 dB have been measured, giving ample headroom to compensate thermoregulatory effects in in vivo tests and showing potential to be adapted for small animal applications, also thanks to the possibility of adjusting, as desired, the maximum temperature achieved and the exposure time. Preliminary studies on 3D cell cultures have already demonstrated the effectiveness of the RF applicator in guaranteeing a good heating uniformity and stability also along in vitro tests, which will be further explored as a future research direction.

## Figures and Tables

**Table 1:** Electrical and thermal properties of the materials considered in the simulations.

Material	*ρ* [kg/m^3^]	*σ* [S/m]	*ε*_r_ ^1^	*C*_p_ [J/(kg·K)]	*k* [W/(m·K)]
HDPE ^1^	950	0.00001	2.1	1900	0.5
PVC ^1^	1380	0.0001	3	1250	0.2
Agar gel	1000	0.755	74	4181	0.563

^1^*ε*_r_ stands for relative permittivity, HDPE for high-density polyethylene and PVC for polyvinyl chloride.

## Data Availability

The data presented in this study will be openly available in Zenodo at DOI 10.5281/zenodo.6410802.

## Supplementary Materials

The following supporting information can be downloaded at: https://www.mdpi.com/article/10.3390/s22103610/s1, Figure S1: Spatial variation of SAR in the phantom calculated along the radial and longitudinal axes, fixing the diameter of the inner conductor to 2.2 cm and 4.3 cm.; Figure S2: Heating-cooling transients as a function of supplied power levels, measured in points #1, #5, #6, #7, #8, #10, #11, #13 and #14; Figure S3: Picture of the split phantom for the measurement of temperature spatial distribution and temperature map directly acquired with IR thermal camera on the central longitudinal plane of the split phantom.
