# Supplementary material for: Design and Characterization of an RF Applicator for In Vitro Tests of Electromagnetic Hyperthermia
Source: Sensors (Basel). 2022 May 10;22(10):3610. doi: 10.3390/s22103610 (PMC9148047; doi:10.3390/s22103610)
Supplement: Supplementary file 1 [file sensors-22-03610-s001.zip › sensors-1696999-supplementary.pdf]

Supplementary Material

Design and characterization of an RF applicator for in vitro tests of electromagnetic hyperthermia

by Riccardo Ferrero, Ioannis Androulakis, Luca Martino, Robin Nadar, Gerard van Rhoon and Alessandra Manzin

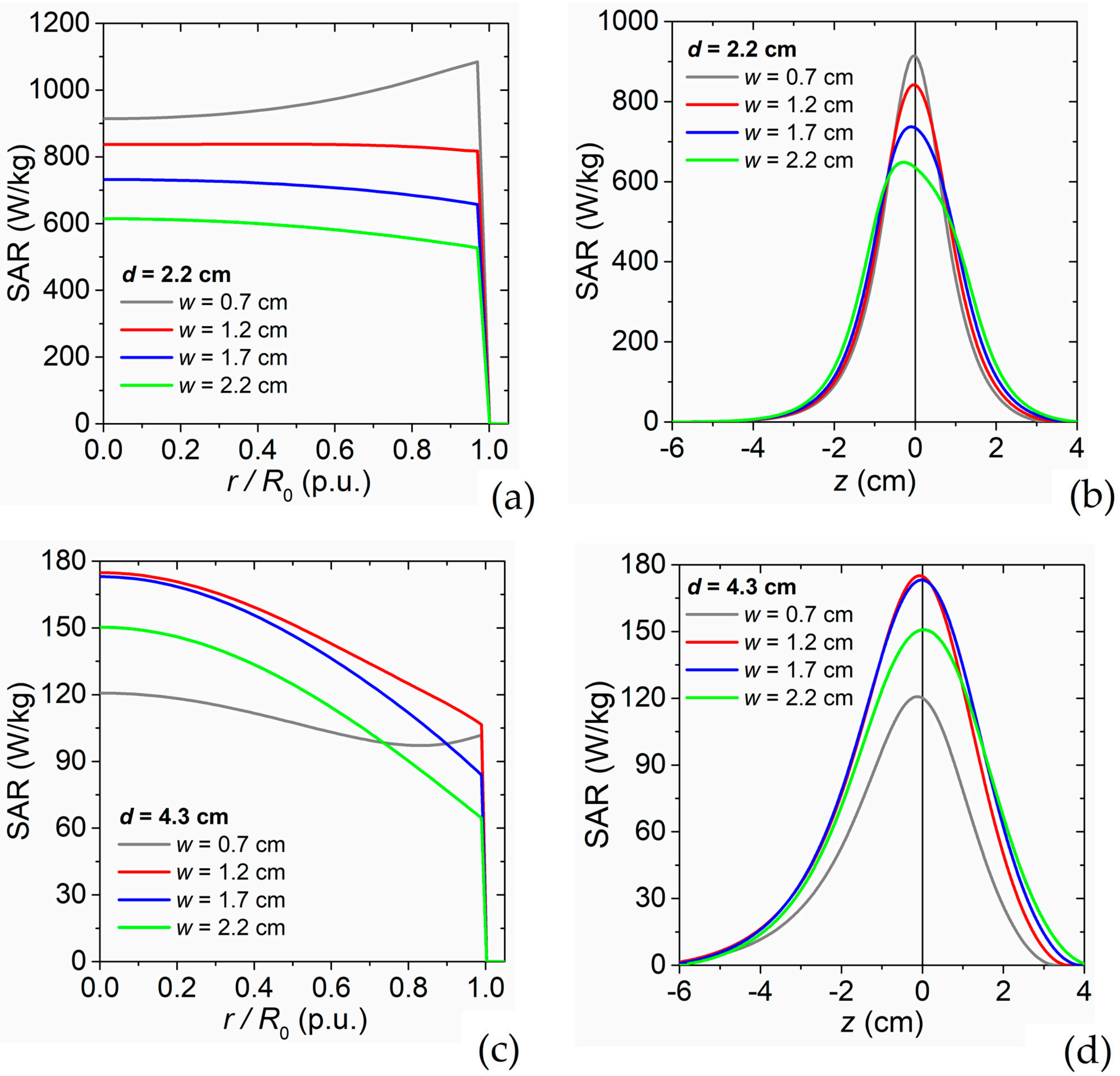

Figure S1. Spatial variation of SAR in the phantom calculated along the radial and longitudinal axes depicted in the schematic of Figure 1c. The calculations are performed for variable values of the aperture width *w*, fixing the diameter *d* of the inner conductor to **(a)**, **(b)** 2.2 cm and **(c)**, **(d)** 4.3 cm.

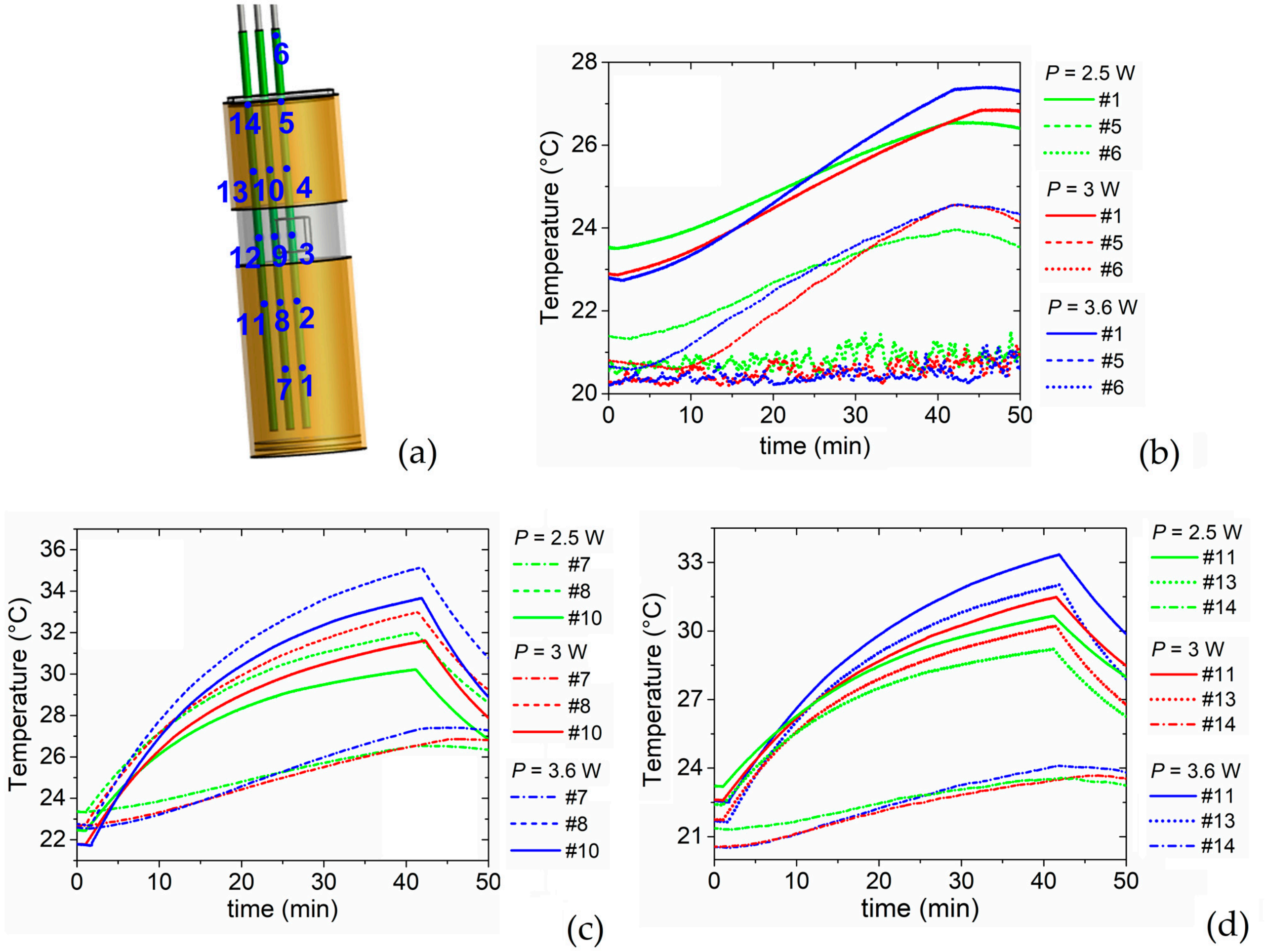

Figure S2. (a) Schematics of the phantom, showing the position of the 14 measurement points distributed along the fiber-optic thermometers. Heating-cooling transients as a function of supplied power levels, measured in points **(b)** #1, #5 and #6, **(c)** #7, #8 and #10, and **(d)** #11, #13 and #14.

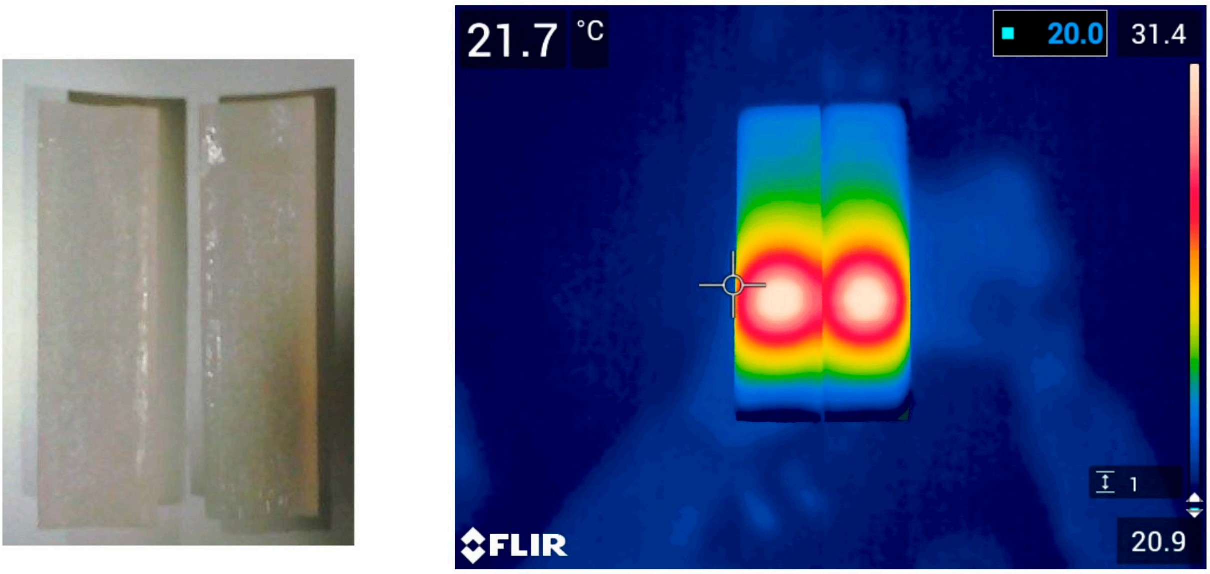

(a) (b)

Figure S3. (a) Picture of the split phantom for the measurement of temperature spatial distribution. **(b)** Temperature map directly acquired with IR thermal camera on the central longitudinal plane of the split phantom, 3 min after heating the phantom with a 5 W power for 5 min.
